# Capturing the embryonic stages of self-assembly - design rules for molecular computation

**DOI:** 10.1038/srep10116

**Published:** 2015-05-11

**Authors:** Peter N. Nirmalraj, Damien Thompson, Heike E. Riel

**Affiliations:** 1 IBM Research–Zurich, Säumerstrasse 4, CH- 8803, Rüschlikon, Switzerland; 2 Materials and Surface Science Institute and Department of Physics and Energy, University of Limerick, Ireland

## Abstract

The drive towards organic computing is gaining momentum. Interestingly, the building blocks for such architectures is based on molecular ensembles extending from nucleic acids to synthetic molecules. Advancement in this direction requires devising precise nanoscopic experiments and model calculations to decipher the mechanisms governing the integration of a large number of molecules over time at room-temperature. Here, we report on ultrahigh-resolution scanning tunnelling microscopic measurements to register the motion of molecules in the absence of external stimulus in liquid medium. We observe the collective behavior of individual molecules within a swarm which constantly iterate their position to attain an energetically favourable site. Our approach provides a consistent pathway to register molecular self-assembly in sequential steps from visualising thermodynamically driven repair of defects up until the formation of a stable two-dimensional configuration. These elemental findings on molecular surface dynamics, self-repair and intermolecular kinetic pathways rationalised by atom-scale simulations can be explored for developing new models in algorithmic self-assembly to realisation of evolvable hardware.

The interaction of simple individual units within an ensemble that leads to advanced collective behavior without any chain of control is generally referred to as swarm intelligence 1,2. This is a highly valued process observed in social insects such as ants^3^,^4^,^5^, which forage for food by finding the shortest route between their nest and the food source after multiple iterations, using an autocatalytic process mediated by a chemical trail. Likewise, bees in a hive exhibit collective behavior, where the scout bee travels extensive distances and then effectively communicates the location of the nectar source with its counterparts through a waggle dance. Such an approach for distributed problem solving has inspired alternative approaches in data processing and automated reasoning through the development of ant colony optimization 1,3,6,7,8, particle swarm optimization^9^ and artificial bee colony optimization algorithms^10^. These nature inspired algorithms have vast opportunities beyond parallel computing to rerouting of communication networks^11^, predicting stock market trends^12^ and in brain image analysis^13^. A common trait in the group behavior of the social insects is stigmery, which is the indirect communication between individual elements in engineering intelligent and ordered structures.

Similar to the self-organised structures at the macroscale, unsupervised ordering also takes place at molecular length scales, driven by the process of self-assembly^14^,^15^,^16^ where molecules interact through attractive and repulsive forces within a population to form ordered structures. This process of self-assembly occurs as the molecules strive to reach an equilibrium state by minimization of free-energy in the closed system, which is in contrast to self-organised structures in the macroscale where energy is dissipated in an open system. The degree of interaction between the individual molecular building blocks and their nearest neighbours can vary from weak van der Waals, hydrogen bonding to π-π stacking contacts. In their eagerness to self-assemble the molecules undergo varied physical processes from dynamic re-organisation, defect-healing within the molecular matrix, ability to co-ordinate in obtaining effective pathways to form ordered domains and Oswald ripening where larger domains are formed due to the coalescence of smaller domains^17^. In contrast to observing stigmeric behavior of social insects which is video recorded digitally in frames per second^4^,^5^, the visualization of molecular motion at the nanoscale requires powerful imaging techniques such as scanning tunnelling microscopy (STM)^18^ which can resolve structure and motion of low dimensional materials from molecules^19^ to atoms^20^ and can operate both in vacuum^21^ and in liquids^16^,^22^,^23^,^24^,^25^,^26^,^27^. While highly ordered two-dimensional molecular structures and their formation mechanisms have been investigated under diverse environments (ultra-high vacuum at cryogenic conditions and in liquids) using STM^28^,^29^,^30^ these studies have focussed on thermodynamically stable architectures where the epitaxial relationship between the adlayer and the surface was the focal point of interest.

The subject matter that remains to be fully clarified is that of the mechanisms leading to the formation of a self-assembled molecular architecture in real-time under innate conditions at room-temperature. This is of significant research interest as knowing the stepwise evolution of molecular systems and pattern formation can serve in modelling algorithms that can be explored in effectively predicting design of new materials formed through self-assembly. Additionally, such information can have broad implications in sectors where bottom-up self-assembly plays a crucial role such as biomolecular computation^31^. However, the practical limitation in establishing a nanoscopic understanding of such dynamic processes lies in striking the precise balance between high spatial and temporal resolution without interfering in the process and taking into account the solvent dynamical effects^16^ during real-time experiments.

In this article we report on the application of a high resolution *in situ* probe microscope capable of capturing stunning real-space snaphots and time-elapsed images of fullerene molecular dynamics at the liquid-metal interface at room-temperature. We observe the local dynamic relocation of individual fullerene molecules and subtle differences in molecular motion as a function of their respective position within the swarm. Crucially, we were able to provide direct experimental evidence for defect formation and healing at a molecular scale. We further rely on atomic-scale molecular dynamics (MD) simulations to compute the intermolecular and metal-molecule interaction energies and quantify the differences in molecular motion as a function of their respective position within the matrix. There is a clear thermodynamic benefit for self-assembly into the observed automatically-healed and tightly-packed patterns. These preliminary design rules gleaned from precise nanoscopic measurements and theoretical calculations can be directly applied in designing new algorithms and refining if possible previously reported step-by-step equilibration rules framed for agent-based algorithms^32^,^33^ and tile assembly model^34^. Such detailed understanding of the governing rules dictating the behavior of molecular swarms will enable the capability to program artificial patterns^35^ based on deterministic swarming of molecules.

## Results and Discussion

**Figure 1:**
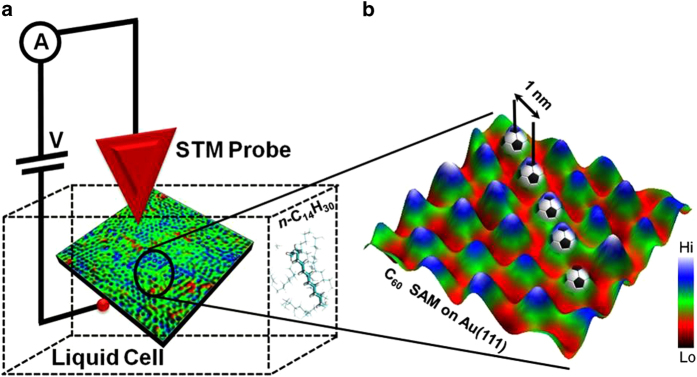
Liquid-STM design for probing molecular self-assembly. (**a**) Schematic of the *in situ* STM setup used to probe self-assembly of C_60_ units. The self-assembled C_60_ molecular layer formed on Au(111) is placed within a compact Teflon-based liquid cell. The *in situ* STM imaging is performed at room-temperature under *n*-tetradecane (*n*-C_14_H_30_) liquid medium (chemical structure shown in the inset) with a metal (Pt/Ir) probe. (**b**) Zoom-in *in-situ* STM image of a well-ordered fullerene molecular layer (C_60_ self-assembled monolayer) on Au(111) recorded in the presence of *n*-tetradecane solvent (Tunnelling set point: I = 3 pA, V = 0.1 V, scan size: 5 nm × 5 nm). The intercage distance between nearest lying fullerne buckyballs is ~1 nm.

A schematic description of the liquid-cell within which the STM imaging measurements on a fullerene (C_60_) molecular layer were performed is shown in Fig. 1a. Extremely low tunnel current setpoints ranging in the order of 2-10 pA is maintained to prevent tip induced rearrangements in the molecular layer or influencing molecular motion. A well ordered and closely packed self assembled molecular layer (SAM) of the C_60_ molecules with an intermolecular distance (measured between the center of one fullerene cage to the other) of ~1 nm is shown in the zoomed-in three-dimensional *in situ* STM image (recorded 10 minutes after drop casting the C_60_ solution on the gold surface). Previously it has been shown that it is possible to trigger a cascading electrical response through a well-ordered molecular layer by applying an input voltage pulse at a specific location over a single molecule within the layer, opening up possibilities for programming molecular circuits^36^. Significant progress towards this goal was recently made when self assembled molecular bi-layers composed of 2,3-dichloro-5,6-dicyano-p-benzoquinone molecules adsorbed on Au(111) were programmed using an STM probe^37^. These electronically activated molecular layers showed switching between multiple conducting states that led to the formation of local circuits within the molecular domain and was further exploited in solving logic gates, calculating Voronoi diagrams, simulating heat diffusion and growth of cancer cells^37^.

**Figure 2:**
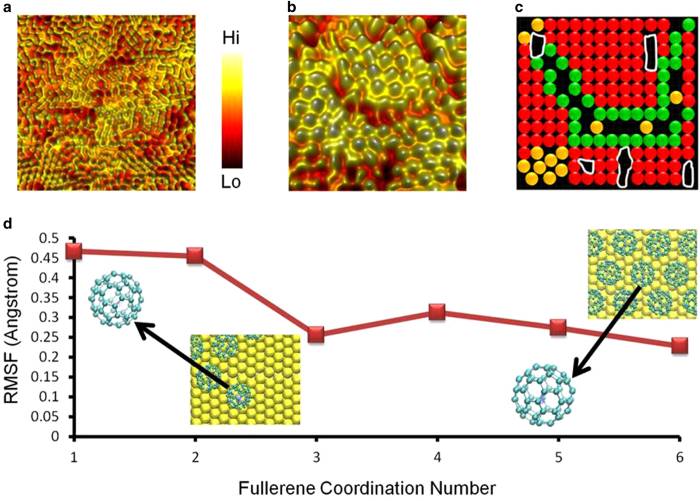
Visualising local molecular interactions within domains. (**a**) Large-area three-dimensionally represented *in situ* STM snapshot showing the arrangement of individual C_60_ units in ordered and disordered regions (Tunnelling set point: I = 5 pA, V = 0.2 V, scan size: 28 nm × 28 nm). (**b**) Spatially magnified image within a region of the molecular layer area shown in panel a (Tunnelling set point: I = 5 pA, V = 0.3 V, scan size: 12 nm × 12 nm). (**c**) Schematic representation of arrangement of fullerene units as depicted in the real-time image in panel b, ordered within the domain (red spheres), at the domain edge (green spheres) and the disordered molecular regions (orange spheres). The white regions indicate the surface defects on the metal surface. (**d**) Values of root mean square fluctuations (RMSF) in positions as a function of C_60_ coordination number, computed using molecular dynamics (MD) simulations. Molecule- and time-averaged uncertainties are 0.01-0.06 Å. Inset panels show representative packing arrangements with C_60_ coordination numbers of 1 (left) and 6 (right) and zoom-in of the molecule include a time-map of center of mass positions during 10 ns of dynamics.

In the present work we unravel the packing of a thermodynamically stable molecular layer with high-spatial resolution but specifically focus on mechanisms dictating the early-phase of molecular self assembly. In particular, we capture the formation of multiple interconnections between the molecules as they form an energetically stable molecular layer at the liquid-solid electrical interface. Figure 2a shows a wide-angle *in situ* STM snapshot (recorded immediately after deposition on gold thin film supported on mica substrate) of C_60_ molecules in three dimensional format showing regions which are partially ordered and the majority of the molecules in a disordered phase as a result of competitive adsorption mechanisms. Zooming-in on a selected region shows well resolved single C_60_ molecular units within local domains (Fig. 2b). Such high-resolution real-space images provide detailed description of single molecular structure, local ordering, intermolecular interactions and the presence of underlying surface defects and their role during molecular self assembly. We have previously examined in detail the specific role of surface defects in hampering molecular self-assembly at the liquid-solid interface and provided solutions to engineer defect-free metal substrates that can serve as ideal platforms to host two-dimensional organic layers^25^. Figure 2b reveals the differences in molecular ordering within a localised region. Based on several *in situ* STM snapshots and time-elapsed imaging we observed the molecules within a domain (as shown in Fig. 2b) to remain stable and less mobile when compared to their counterparts located at the edges of the local domains. The ball model shown in Fig. 2c emulates the *in situ* STM image (Fig. 2b) with the stable molecules (marked in red) within the domains, the high-mobility molecules in the periphery (marked in green), disordered molecules (marked in orange) and the location of the underlying surface defects (coded in white) that remain unfilled by the molecules. Our experimental observations on molecular mobilities are consistent with previous *in situ* STM studies on isolated C_60_ molecular islands^38^,^39^.

It is worth mentioning that the observed behaviour of single molecules within a closely packed population bear resemblance to a certain extent the flocking mechanism at the macroscale (in birds and herding animals) which follows the geometry of the selfish herd^40^, where the individuals at the periphery of the flock are more mobile and at greater risk than their counterparts in the centre, labelled as low-risk central positions within a specific domain.

To better understand and quantitatively map the location-specific differences in single molecule mobility, we performed molecular dynamics (MD) simulations taking into account the actual experimental conditions (many C_60_ molecules assembling on Au(111) in bulk solvent medium). From the simulation cells (see Supplementary Information for details), the root mean square fluctuations (RMSF) in C_60_ location are calculated over 100 equally-spaced molecular structures sampled during the final 10 nanoseconds (ns) of 30 ns of room temperature dynamics. Figure 2d shows a plot of RMSF as a function of the fullerene coordination number, where the RMSF gradually decreases as the C_60_ coordination number is increased in steps. The RMSF values are computed from the centres of mass of each C_60_ molecule and the values are averaged over multiple molecules (binned according to C_60_ coordination number) and temporal structures. The simulation data indicates that the low-coordination species are more mobile than buried species, thereby substantiating the STM findings.

**Figure 3:**
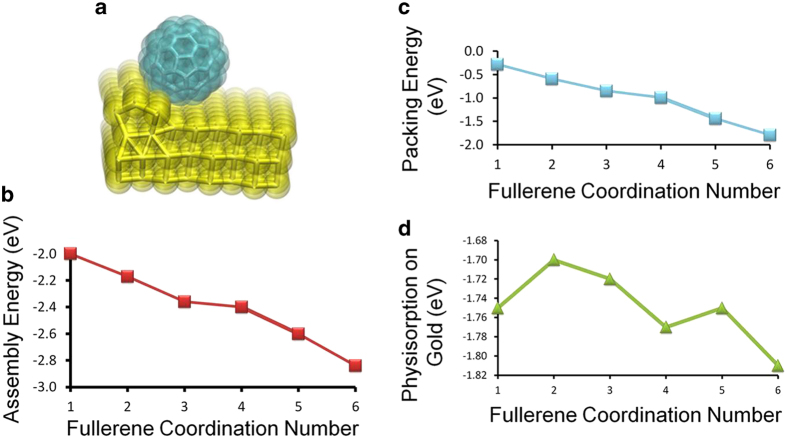
Calculation of C_60_-gold interactions (**a**) The single-molecule C_60_-gold complex computed using dispersion corrected density functional theory (DFT). The physisorption energy is calculated relative to a control simulation of a completely desorbed C_60_ and bare gold surface and agrees almost perfectly with the physisorption energy calculated from the MD force field shown in panel d . (**b**) Assembly energy is summed over C_60_-C_60_ packing energy (panel **c**), C_60_-gold physisorption (panel **d**) and C_60_ desolvation penalty in *n*-C_14_H_30_ (which increases from 0.1 to 0.8 eV as C_60_ coordination number increases from 1 to 6. Time- and structure-averaged errors for packing and physisorption energies are 0.05 eV and 0.14 eV. Packing becomes similar in magnitude to physisorption once a hexagonally close packed arrangement is assembled. All energies are reported in eV per C_60_ molecule.

However, irrespective of the number of nearest neighbours available for the molecules, the interaction between the molecules and the underlying gold atomic lattice needs to be understood. For this we rely on van der Waals corrected density functional theory (DFT)^41^ to compute the C_60_-Au physisorption energy. Figure 3a shows the corresponding electronic structure of the metal-organic complex for which we calculate a total binding energy of 1.77 eV. This interaction energy is similar to the strength of the alkanethiol-gold bond^42^,^43^ and sufficient to anchor the C_60_ molecules on the surface whilst permitting the molecules to move in two-dimensional space to form a thermodynamically stable and close packed molecular film. Another parameter that provides information on intermolecular mechanics is the assembly energy, which is the sum of the packing energy between neighbouring C_60_ molecules (Fig. 3c) and the C_60_-gold physisorption energy (Fig. 3d). Time- and structure-averaged errors for packing and physisorption energies are 0.05 eV and 0.14 eV. A gradual downward shift in the assembly enthalpy from −2 eV to −2.8 eV is computed for a fullerene molecular layer as the C_60_ coordination number is incremented from 1 to 6. This clearly highlights the energetic payoff for formation of the hexagonally close packed structure observed in the STM experiments. By comparison, entropic effects are very small (−TΔS = −0.04 ± 0.03 eV at room-temperature averaged over all molecule coordination numbers, computed using the method of Schlitter^44^,^45^ to extract entropy values from MD trajectories). In addition to the high-resolution snapshots of the closely packed, fully-ordered (Fig. 1a,b) and partially-ordered (Fig. 2a,b) molecular domains, we were able to resolve in real-time the formation of defects within the molecular layer and the repair of these defects over time.

**Figure 4:**
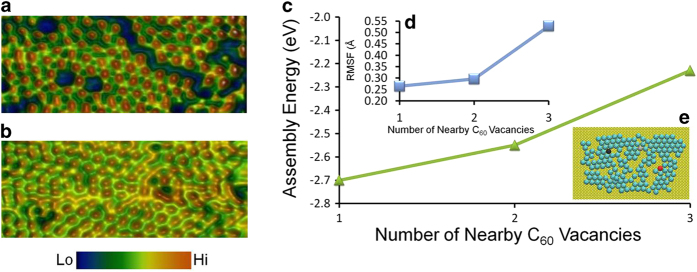
Defect-healing within an organic matrix (**a**) and (**b**) Time-elapsed *in situ* STM imaging of C_60_ molecular layers showing defect-healing over time. (**a**, imaged immediately after drop-casting molecular solution, **b**, imaged 5 seconds after recording the molecular dynamics snapshot in a). (Tunnelling set point: I = 8 pA, V = 0.4 V, scan size: 8 nm × 20 nm). (**c**) Plot of assembly energy of the molecular film and RMSF (panel **d**) as a function of nearest C_60_ vacancies. Assembly energies are reduced in magnitude as the size of neighbouring holes in the SAM increases. (**e**) Snapshot from a simulation cell, where all three molecules highlighted have nearest-neighbour coordination numbers of 5 but their assembly energies reflect their longer range environment. This increased disorder near holes is reflected also in the increased mobility of fullerene molecules near holes as measured by RMSF fluctuations in computed C_60_ centers of mass. Both packing energy and physisorption energies are smaller in magnitude near larger holes, which provides the thermodynamic driver towards filling-in and repair of holes in the assembling layer.

A high-resolution *in situ* STM image of a C_60_ molecular layer recorded immediately after liquid-phase deposition on gold thin film supported on halide substrate (see methods section for sample preparation) is shown in Fig. 4a. Distinct regions of voids (unfilled areas, color coded in blue) is visible in between the molecular ensembles showing local ordering. Upon imaging the same region after a time-elapse of 5 seconds (Fig. 4b) the previously observed defects were no longer visible after the arrival of new molecules and through dynamic rearrangement of the previously available local molecules. Furthermore, an overall change in the orientation of the molecular units within the colony is seen from the time-elapsed image (Fig. 4b). The MD structures reveal that the assembly energies are reduced in magnitude as the size of neighbouring holes in the molecular layer increases (Fig. 4c). All three molecules highlighted have nearest-neighbour coordination numbers of 5 (Fig. 4e) but their assembly energies reflect their longer range, up to next-nearest neighbour, environment (Fig. 4c). This destabilisation near holes in the film is reflected in the increased mobility of C_60_ molecules near holes as measured by RMSF fluctuations in computed C_60_ positions (Fig. 4d). In general the combined real-space information and simulation dataset indicates that the buried molecules in the center of the colony are more ordered and energetically stable than their counterparts in the edges.

**Figure 5:**
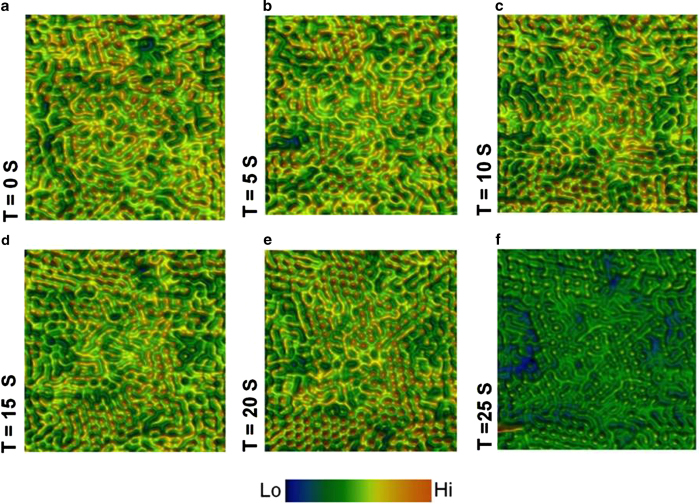
Evolution of a thermodynamically stable molecular configuration (Tunnelling set point: I = 10 pA, V = 0.3 V, scan size: 22 nm × 22 nm). (**a**)–(**f**) Series of wide-angle real-time *in situ* STM images over a fixed region of C_60_ molecular layer that progresses from a disordered (a) to an ordered phase (f). The time-elapse between each frame is 5 seconds.

Capturing minute structural fluctuations in the cooperative behaviour of molecules and registering features such as molecular error correction is experimentally challenging especially at the liquid-solid interface^22^,^29^,^39^,^46^. As can be seen in Fig. 5(a–f) the step-by-step evolution of an initially disordered molecular layer with individual molecules attempting to self-assemble through weak intermolecular bonding to form ordered and compact molecular architectures (molecular layers formed on gold thin films supported on halide platform). The time-elapse between each *in situ* STM frame is 5 seconds which is the maximum optimal scanning speed limit where relevant information on both molecular structure and local dynamics can be obtained using our experimental design. Higher scanning speeds can be achieved (~20 frames/second) and data can be recorded closer to video-rates by upgrading the electronics design (bandwidth of power amplifier and feedback control system) of the scanning probe microscope and achieving suitable signal-to-noise ratio^47^. The time-elapsed images show that the molecular swarms to heal defects where the defect of one molecular element does not influence the end result as it is compensated by the rest of the functional molecular units. This experimentally observed behaviour without the influence of external stimulus detailing the local interaction between electronic elements which are influenced only by their local nanoscopic environment and not from far-lying components forms the first step towards the realisation of the previously theoretically predicted concept of evolving hardware^48^.

**Figure 6:**
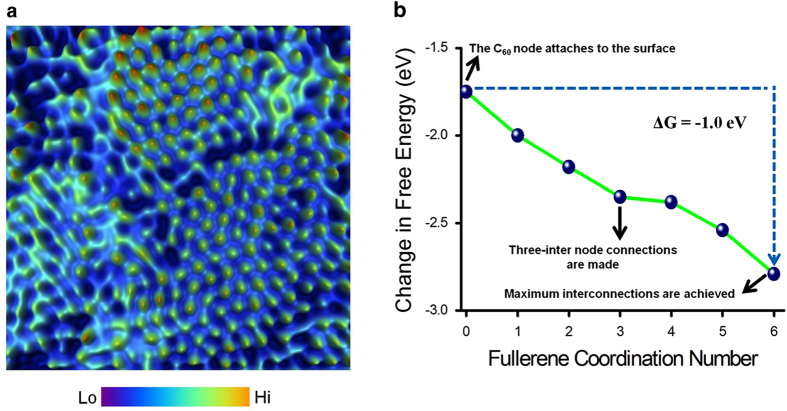
Free energy of self-assembled fullerene molecular layer (a) High-resolution three-dimensionally represented *in situ* STM snapshot of a fullerene molecular layer with different packing order between certain molecular segments within a single-frame (Tunnelling set point: I = 2 pA, V = 0.5 V, scan size: 20 nm × 20 nm). (b) Plot of Gibbs free energy of the molecular layer as a function of the fullerene coordination number. The line is drawn to guide the eye and indicates a net free energy benefit of 1.8 eV for sticking a C_60_ node to the substrate plus an additional 1.0 eV for wiring up the C_60_ node by connecting to six neighbouring nodes with an average benefit of -0.2 ± 0.1 eV for making each additional connection from 0 to 6. Note that a steeper curve is obtained if the desolvation penalties are not included (which would approximate a film transferred to ultra high vacuum), as shown in Supplementary Information.

Figure 6a is a high-resolution three-dimensionally represented *in-situ* STM image of a molecular layer showing the arrangement of individual molecules within the matrix and areas adjacent to the molecules that remain empty. The computed free energy profile in Fig. 6b was generated by summing the enthalpy and entropic terms described earlier. The profile shows the net free energy benefit of approximately 1.8 eV for adsorption of a single C_60_ molecule to Au(111) plus an additional 1.0 eV that is obtained from intermolecular packing, as the C_60_ node is connected to six neighbouring nodes. This thermodynamic driver towards pristine SAMs is due purely to C_60_-C_60_ van der Waals packing interactions. The plot shows an average benefit of −0.2 ± 0.1 eV obtained for each additional contact made as the C_60_ goes from an isolated adsorbate (coordination number of zero) to a maximally coordinated (coordination number of six) node in the hexagonally-close packed monolayer. Note the free energy benefit becomes small in the middle of cluster-formation (ΔG = 0.03 eV for increasing the coordination number from 3 to 4, Fig.6b) and then becomes large again (−0.25 eV) as the final connection is made to give the maximum coordination number of 6. This transition may explain the “lock down” observed in some of the STM data. As shown in Fig. 5 the more ordered assemblies that form over time typically have shorter C_60_-gold distances as well as shorter C_60_-C_60_ contacts.

## Outlook

The nascent stages of molecular self assembly and the governing dynamics of this process in forming energetically favourable configurations is registered through high-resolution tunnelling microscopy. These studies at a single-molecule level were conducted at the liquid-solid interface under room-temperature conditions. The benefits of capturing step-by-step processes involved in active two-dimensional self assembly of molecular components under ambient conditions is clearly highlighted. The ability of a molecular layer to heal defects without any central organization is recorded in real-time. Self-repairing molecular circuits is a highly desired feature in next-generation computing, as it provides the unique possibility to develop fault-tolerant circuits.

The organic elements were observed from the STM time-elapsed data to have variations in their respective mobilities as a function of their position within the domain. These observations are further substantiated by unbiased atom-scale simulations, thus emulating a molecular swarm. The intriguing mechanisms associated with the formation and functioning of the molecular swarms can be useful in providing valuable insights when developing future mathematical models capable of predicting how a complex system of disorganized elements coordinate locally to form organised patterns, with minimal participating elements and in short sequential steps. These observations into early-stage SAM formation will also be constructive for validating and fine-tuning the empirical force fields and dispersion corrections being developed to supplement quantum mechanics in the rational design of nanomaterials. The STM images provide reference interaction lengths for the parameterisation of molecule-surface and molecule-molecule interactions.

On a parallel track, the insights obtained into the molecular structure, dynamics and two-dimensional network formation on surfaces can be used as reliable inputs in writing logical programs for visualising the growth of nanostructures. We expect that it will also serve as useful guidelines for future programming of artificial nanomaterials composed of biomolecular systems^49^,^50^,^51^,^52^ and can form the basis for designing operating protocols for self-assembling collective machines inspired by nature^53^ to origami^54^.

## Methods

### Molecular solution and metal substrate preparation

The C_60_ molecules (99% purity) were purchased from Alfa Aesar in powder form and dispersed in *n*-tetradecane solvent (Sigma Aldrich) at a concentration of 10^−2^ M using a low-power sonic bath procedure for ten minutes followed by centrifugation at 1500 rpm for 60 minutes. 10 μL of the as-prepared supernatant C_60_ solution was gently drop casted onto the gold thin film, placed within a Teflon-based liquid-cell for subsequent STM imaging at room-temperature. For the metal substrate 100 nm of Au (99.99%) was electron-beam evaporated (BOC Edwards, Chamber Pressure 10^−6^ mbar) onto freshly cleaved mica (High quality Grade V1, muscovite mica, purchased from SPI Supplies) at a rate of 1 Å s^−1^. Such metal thin films on mica were found to contain nanoscale defects that impeded molecular self-assembly. For engineering defect-free and ultra flat gold thin films, we employ halide support platforms on which gold was evaporated using identical protocols as reported previously^25^.

### Liquid-STM measurement protocol

The STM measurements were performed in constant-current mode at room-temperature using a Veeco Scanning Tunnelling Microscope, Nanoscope IIIa, Multimode (Scanner model: E- Scanner). A mechanically cut Pt/Ir wire (0.25 mm, Good Fellow GmbH) was used as the STM probe. For real-time tracking of molecular dynamics the STM probe was scanned at a speed of 1 ms/line with a tip drift rate of ~1 nm/min in *n*-tetradecane liquid medium. The *in situ* STM measurements were performed in state-of-the-art noise free laboratories with excellent noise, vibration, temperature and humidity control. The bias voltage is applied to the sample and the metal tip is grounded through the high-sensitive preamplifier. The pre-amplifier employed in this study is capable of current detection as low as 1pA. Image processing was performed using Gwyddion 2.39. To avoid tip artifacts the STM metal probe was calibrated by measuring standard reference differential conductance spectra and imaging over blank Au(111) surface.

### Modelling and theory calculations

The C_60_ physisorption energies on Au(111) were calculated using two independent methods, van der Waals corrected DFT^41^ and empirical force fields^55^. C_60_ monolayer assembly was modelled using molecular dynamics simulations. Full details of the methods and models used are given in the supporting information.

## Additional Information

**How to cite this article**: Nirmalraj, P. N. *et al*. Capturing the embryonic stages of self-assembly - design rules for molecular computation. *Sci. Rep.* **5**, 10116; doi: 10.1038/srep10116 (2015).

## Supplementary Material

Supporting Information

Supporting Information

Supporting Information

Supporting Information
