# Supplementary material for: Capturing the embryonic stages of self-assembly - design rules for molecular computation
Source: Sci Rep. 2015 May 11;5:10116. doi: 10.1038/srep10116 (PMC4650799; doi:10.1038/srep10116)
Supplement: Supporting Information [file srep10116-s4.pdf]

Capturing the embryonic stages of self-assembly - design rules for molecular computation.

Peter N. Nirmalraj*, Damien Thompson and Heike E. Riel

* To whom correspondence should be addressed: pni@zurich.ibm.com. Tel: +41-447248875

Full details of computer models and simulation protocol

All simulation files are available on request from DT (damien.thompson@ul.ie)

1. Van der Waals corrected electronic structure calculations of C_{60} physisorption on Au(111)

The C_{60} physisorption energies on Au(111) are calculated using two independent methods.

The van der Waals contribution to adsorption is calculated from the Grimme D3 correction[1] to DFT electronic structure calculations using the VASP program[2] to determine the molecular adsorption complex of C_{60} on Au(111). The periodic surface models (Fig. 3a) were described using periodic plane wave DFT with the GGA-PBE functional[3], projector augmented wave (PAW) pseudopotentials[4] with a plane wave cut-off of 400 eV and a vacuum spacing of approximately 4 nm in the direction normal to the gold surface. The molecule-surface complexes were calculated using periodic boundary conditions. Molecule-surface binding energies were converged to below 100 meV using a 441 k-point grid. The van der Waals contribution is estimated by calculating dispersion interactions within a cut-off of 12 Angstrom (reducing the cutoff to 5 Angstrom slightly reduces the vdW correction to -1.58 eV) and normalising the vdW energy relative to a control simulation cell in which the molecule and surface are completely separated at a distance of 1.6 nm. This yields a physisorption energy of -1.77 eV. The van der Waals interaction energy is also computed from room temperature molecular dynamics simulations (Fig. 2, and see section 2 below). Averaging over all mono-coordinated C_{60}:Au(111) complexes gives a physisorption energy of -1.75 ± 0.04 eV, in excellent agreement with the D3-DFT value.

2. Film assembly modelled using molecular dynamics simulations

(a) Deposition and island formation

Molecular dynamics simulations of early stage C_{60} SAM formation on Au(111) show competing inter-molecular and molecule-surface interactions that give rise to close packed patterns of C_{60} units at experimental timescales. The bulk liquid sheath is modelled using a cloud of 3750 $C_{14}H_{30}$ molecules (Fig. S1).

Fully atomistic molecular dynamics simulations were performed using the using the NAMD code[5] with the CHARMM potential energy function[6] with additional organic-metal parameters[7]. Simulations were performed at room temperature using a two femtosecond timestep and structures were sampled for 30 nanoseconds of molecular dynamics, following minimisation and two nanoseconds of thermalisation and equilibration. Gold atoms were constrained to their starting crystallographic positions and kept neutral in the simulations.

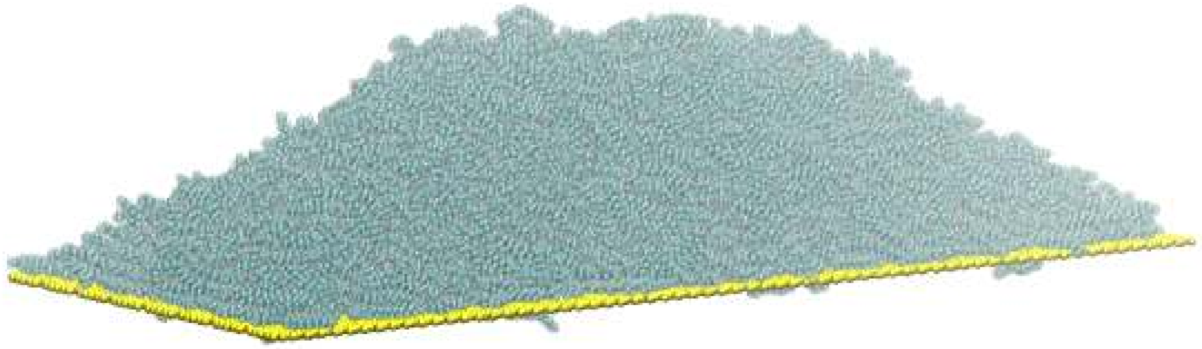

Figure S1. Full simulation cell used to model C_{60} film assembly on Au(111) in bulk n-tetradecane liquid. All image generation and Tcl script-based trajectory analysis was performed using the VMD program[8].

Supplementary movie formation.mpg shows deposition of C_{60} from solution and island formation, over the course of the first 15 ns of dynamics. The first frame is shown below; note the bulk liquid is removed for clarity.

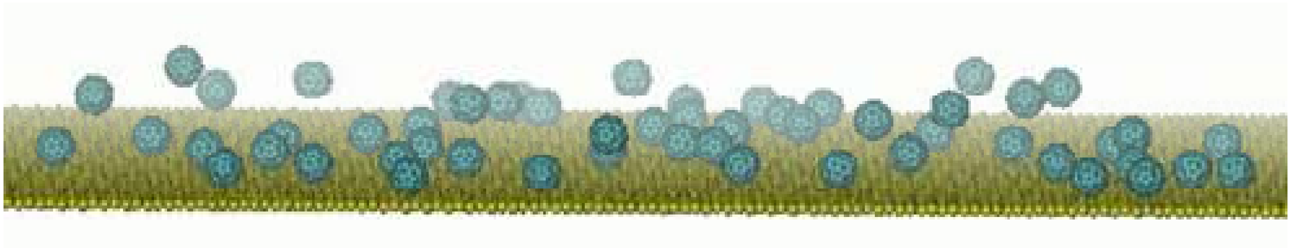

Figure S2. Starting structure used to model C_{60} film assembly on Au(111) in bulk n-tetradecane liquid (liquid omitted for clarity). The final structure formed following 30 ns of equilibrated room temperature molecular dynamics is shown in Fig. 4e.

(b) Large-area molecular film with hexagonally close packed regions and holes linked by low-coordination fingers and bridges.

Supplementary movie equilibrated.mpg shows the damped dynamics of molecules in the film for the final 15 ns of dynamics, due to the combination of vertical sandwiching between the substrate and the liquid, and the progressively stronger horizontal inter-fullerene packing. Holes in the SAM are healed at experimental timescales (Fig. 4b) but remain at the tens of nanoseconds timescale of the simulations, allowing calculation of molecule mobilities/stabilities as a function of coordination number in the evolving SAM. Finally, for the sake of completeness, the free energy profile given in main text Fig. 6 is shown below with a second profile overlaid. This second profile omits in the energy sum the desolvation penalties incurred as C_{60} molecules move from complete solvation in bulk n-$C_{14}H_{30}$ to progressively less-solvated surface-bound states. This alternative model approximates a film that has been transferred from liquid to ultra-high vacuum, and represents the theoretical maximum for C_{60} film self-assembly in which C_{60}-C_{60} connections are made in the absence of competing C_{60}-$C_{14}H_{30}$ interactions.

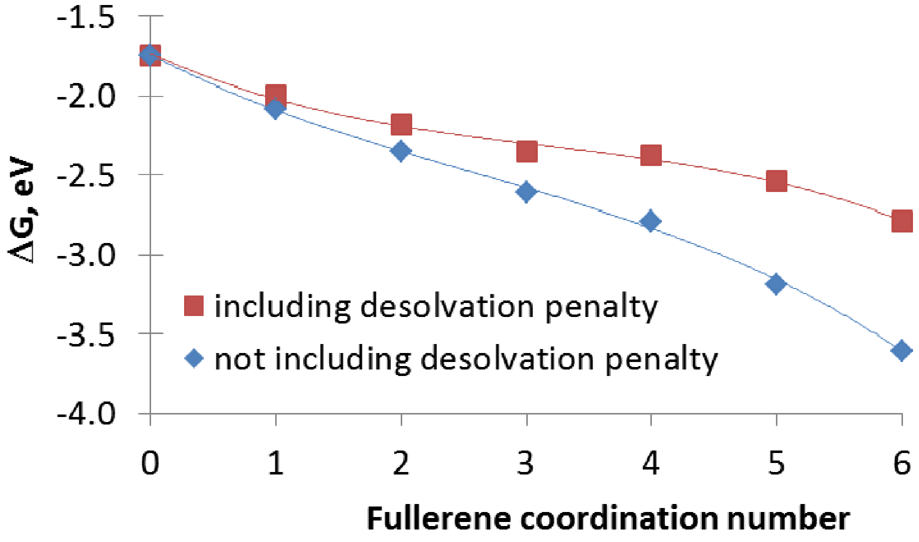

Figure S3. Plot of Gibbs free energy of the molecular layer as a function of the fullerene coordination number, with and without explicit inclusion of C_{60}-$C_{14}H_{30}$ desolvation penalties in the energy sum. Different solvents may offset the C_{60}-C_{60} SAM packing energies to different degrees and shift the effective SAM formation energies in between the two curves

References

[1] S. Grimme, J. Antony, S. Ehrlich, and H. Krieg, "A consistent and accurate ab initio parametrization of density functional dispersion correction (DFT-D) for the 94 elements H-Pu," *Journal of Chemical Physics,* vol. 132, Apr 21 2010.

[2] G. Kresse and J. Hafner, "Abinitio Molecular-Dynamics for Liquid-Metals," *Physical Review B,* vol. 47, pp. 558-561, Jan 1 1993.

[3] J. P. Perdew, K. Burke, and M. Ernzerhof, "Generalized gradient approximation made simple (vol 77, pg 3865, 1996)," *Physical Review Letters,* vol. 78, pp. 1396-1396, Feb 17 1997.

[4] P. E. Blochl, "Projector Augmented-Wave Method," *Physical Review B,* vol. 50, pp. 17953-17979, Dec 15 1994.

[5] J. C. Phillips, R. Braun, W. Wang, J. Gumbart, E. Tajkhorshid, E. Villa*, et al.*, "Scalable molecular dynamics with NAMD," *Journal of Computational Chemistry,* vol. 26, pp. 1781-1802, Dec 2005.

[6] A. D. MacKerell, D. Bashford, M. Bellott, R. L. Dunbrack, J. D. Evanseck, M. J. Field, *et al.*, "All-atom empirical potential for molecular modeling and dynamics studies of proteins," *Journal of Physical Chemistry B,* vol. 102, pp. 3586-3616, Apr 30 1998.

[7] H. Heinz, R. A. Vaia, B. L. Farmer, and R. R. Naik, "Accurate Simulation of Surfaces and Interfaces of Face-Centered Cubic Metals Using 12-6 and 9-6 Lennard-Jones Potentials," *Journal of Physical Chemistry C,* vol. 112, pp. 17281-17290, Nov 6 2008.

[8] W. Humphrey, A. Dalke, and K. Schulten, "VMD: Visual molecular dynamics," *Journal of Molecular Graphics,* vol. 14, pp. 33-&, Feb 1996.
